# Follicular Lymphoma Manifesting as an Isolated Colorectal Polyp‐Like Morphology

**DOI:** 10.1002/jgh3.70170

**Published:** 2025-04-30

**Authors:** Yasuhiko Hamada, Yuhei Umeda, Hayato Nakagawa

**Affiliations:** ^1^ Department of Gastroenterology and Hepatology Mie University Hospital Tsu Japan

**Keywords:** colonoscopy, colorectal polyps, follicular lymphoma

## Abstract

**Background:**

Primary gastrointestinal (GI) follicular lymphoma (FL) is a rare entity, and colorectal FL presenting as a solitary polyp is particularly uncommon.

**Case Presentation:**

A 60‐year‐old woman presented with hematochezia. Colonoscopy incidentally identified an 8‐mm polyp in the transverse colon, which was subsequently removed via cold snare polypectomy. Histopathological examination confirmed Grade 1 FL, with immunohistochemical analysis revealing positivity for CD10, CD20, and Bcl‐2. Further diagnostic evaluation demonstrated FL involvement in the proximal jejunum, leading to a diagnosis of clinical stage I primary GI‐FL. The patient elected to undergo a watch‐and‐wait approach.

**Conclusion:**

This case highlights the potential for colorectal‐FL lesions to be misidentified as adenomatous polyps, posing a diagnostic challenge.

## 1 Introduction

Primary gastrointestinal (GI) follicular lymphoma (FL) is a relatively rare disease, accounting for only 1%–3% of GI lymphomas [1]. Within the GI tract, FLs appear mainly as multiple white nodules in the second portion of the duodenum but may rarely be seen in other parts of the GI tract, such as the colorectum. A study reported 125 patients with primary GI‐FL having duodenal involvement in 89% of cases, while the jejunum was involved in 40% and the ileum in 22%; in contrast, cecal, colonic, and rectal involvement was observed in only 2%, 1%, and 2% of cases, respectively [2].

A previous study categorized the endoscopic appearances of colorectal FL into three subtypes: papular, polypoid, and flat‐elevated types [3]. However, colorectal FL presenting as a single, isolated polyp does not conform to any of these established categories; therefore, such a case is extremely rare [4, 5, 6].

This study reports a case of a colorectal FL presenting as an isolated polyp with a review of the related literature.

## 2 Case Report

A 60‐year‐old woman presented to the gastroenterology clinic due to an episode of hematochezia. She denied any significant personal or family medical history and was not taking any medications. At presentation, her vital signs and physical examination results were unremarkable, and her laboratory test results were within normal ranges. A colonoscopy performed 10 years earlier as part of routine screening had revealed no abnormalities.

**FIGURE 1:**
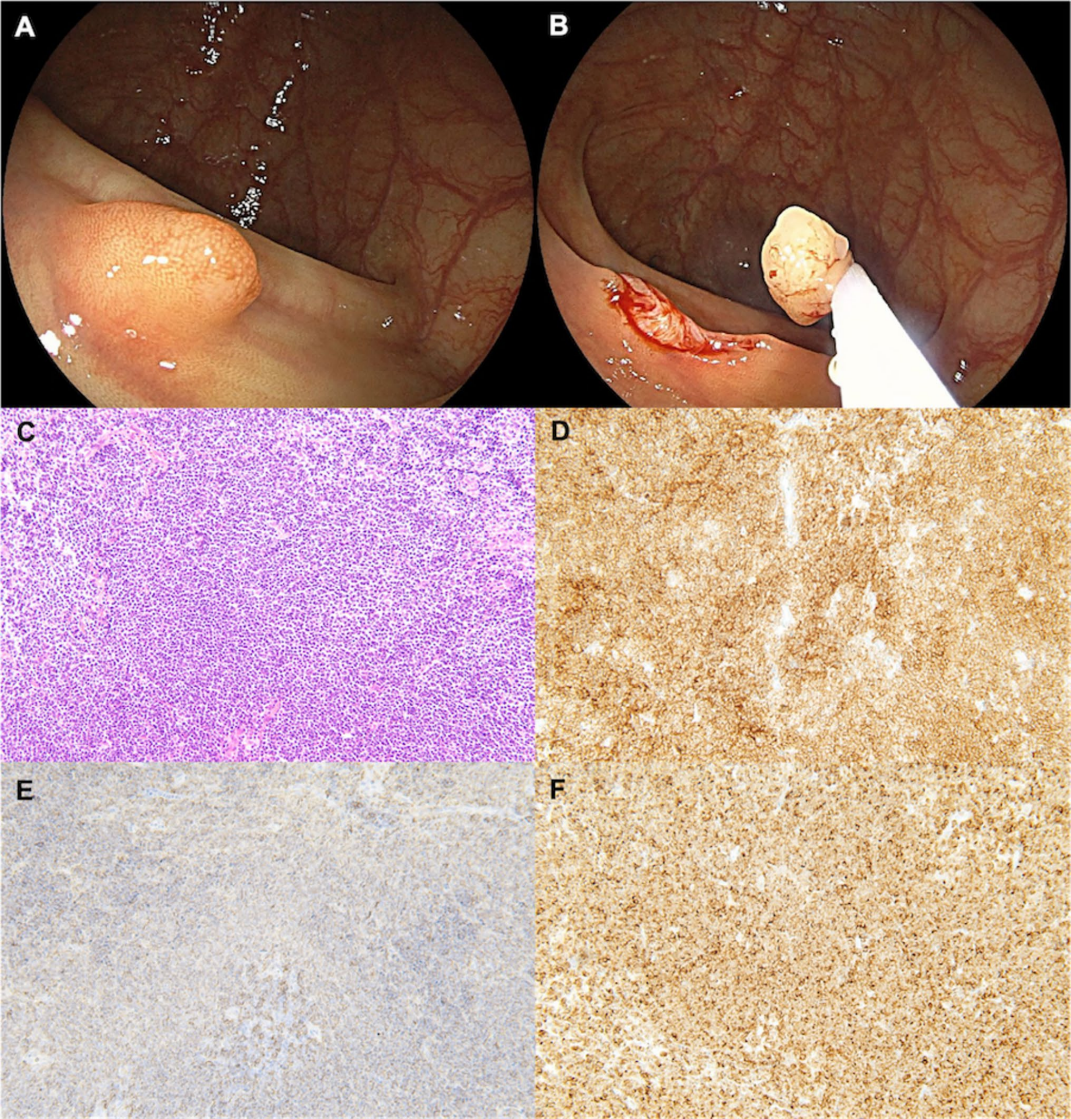
Colonoscopy showing an 8‐mm sessile polyp in the transverse colon. (A) Close‐up view. (B) Removal of the polyp using cold snare polypectomy. (C) Hematoxylin–eosin staining of the resected polyp showing follicular structures containing a proliferation of small abnormal lymphocytes with dense nuclei (original magnification ×200). Immunohistochemical staining of the resected polyp (D: CD10, E: CD20, F: BCL‐2, original magnification ×200) revealed that the lymphocytes were positive for CD10, CD20, and Bcl‐2, consistent with a diagnosis of Grade 1 follicular lymphoma.

A diagnostic colonoscopy performed after the current episode of hematochezia failed to identify the source of the bleeding. However, an 8‐mm sessile polyp was incidentally found in the transverse colon (Figure 1A). Conventional endoscopy showed that the polyp had a slightly reddish appearance without depression. The endoscopist diagnosed the lesion as an adenomatous polyp; the polyp was then removed using cold snare polypectomy (Figure 1B). Histopathological examination of the resected specimen revealed follicular structures with a proliferation of small, abnormal lymphocytes with dense nuclei (Figure 1C). Immunohistochemical staining revealed that the lymphocytes were positive for CD10 (Figure 1D), CD20 (Figure 1E), and Bcl‐2 (Figure 1F), but negative for CD3 and Cyclin D1, consistent with a diagnosis of Grade 1 FL.

Further diagnostic workup, including whole‐body computed tomography and esophagogastroduodenoscopy, showed unremarkable results. Video capsule endoscopy showed multiple white nodules in the proximal jejunum, strongly suspected FL involvement; however, the balloon‐assisted enteroscopy and biopsy for pathological diagnosis could not be performed as the patient did not provide consent. Bone marrow examination revealed no involvement of lymphoma cells. Based on these findings, the patient was diagnosed with primary GI‐FL, clinical stage I, according to the Lugano classification system for GI lymphomas [7]. After discussing treatment options, the patient opted for a watch‐and‐wait approach [8].

## 3 Discussion

The site involved most frequently in GI‐FL is the second portion of the duodenum; in contrast, colorectal involvement is less common in FL cases. In a large series of GI‐FL, Takata et al. reported that, among 125 patients with GI‐FL, colorectal FL occurred in only 0.04% of all cases [2]. Therefore, the endoscopic features of colorectal FL have been reported rarely in the literature.

As mentioned in the introduction section, Iwamuro et al. divided the endoscopic features among 12 colorectal FL cases into 3 subtypes [3]. However, the endoscopic features in our case were rare and did not conform to any of these established categories. Our review of the well‐documented English‐language literature up to 2023 identified only four reported cases of colorectal FL presenting as an isolated polyp, including the current case (DOC. S1) [4, 5, 6]. All four cases were diagnosed at clinical stage I; a watch‐and‐wait approach was adopted for the two cases where detailed follow‐up information was available. Among the cases with well‐documented polyp morphology, the most common type was sessile (2/3, 66.6%). In particular, the FL lesion in the current case was the smallest and most resembled adenomatous polyps in morphology. In all four cases, the FL lesions were initially misdiagnosed and removed as routine adenomatous polyps.

Given this, distinguishing colorectal FL presenting as an isolated polyp from common adenomatous polyps during conventional colonoscopy is a diagnostic challenge, particularly in the absence of meticulous endoscopic evaluation. In the retrospective evaluation, careful observation could have identified that the FL lesions in the four cases manifested a subepithelial rather than epithelial form and did not exhibit the typical findings associated with adenomatous polyps.

Furthermore, previous studies highlighted the utility of magnified observation with narrow‐band imaging for the diagnosis of colorectal FL [9, 10] and revealed distinctive coiled and elongated microvascular patterns and white opaque spots beneath the microvessels, characteristic of FL. In the present case, endoscopic resection was performed without magnifying observation with narrow‐band imaging, which might have prevented the initial misdiagnosis if utilized.

We opted for the watch‐and‐wait approach in the current case. A retrospective cohort study of 73 patients (predominantly Stage I) has reported a 5‐year overall survival of 92.9% and a 10‐year overall survival of 87.1%, with disease progression in 9.6% and spontaneous regression in 16.4% of cases. Takata et al. retrospectively investigated 125 patients with stage I and II intestinal FL [2]. In the median follow‐up period of 3.3 years, 33 patients were followed using the watch‐and‐wait approach: two patients (6.1%) experienced lymphoma progression. None of the patients died of lymphoma. The 5‐year overall survival rate of the 125 patients was 100%, and the 5‐year progression‐free survival rate was 93%. Findings in these studies support a watch‐and‐wait approach for GI‐FL with localized diseases, such as the current case.

In conclusion, this case highlights the importance of careful evaluation of colorectal polyps, especially those that appear isolated and have subepithelial‐like morphology. While rare, FL presenting as an isolated colorectal polyp should be considered in the differential diagnosis to ensure accurate management and prevent misdiagnosis. Careful observation and advanced endoscopic techniques, such as narrow‐band imaging, may prove invaluable in distinguishing FL from adenomatous polyps.

## Ethics Statement

All procedures followed were performed in accordance with the ethical standards of the Declaration of Helsinki and its later amendments.

## Consent

Written informed consent was obtained from the patient for publication of this case report and accompanying images.

## Conflicts of Interest

The authors declare no conflicts of interest.

## Supporting information


**Table S1.** Case series of colorectal follicular lymphoma presenting as a single, isolated polyp (English literature only).
