# Supplementary material for: Follicular Lymphoma Manifesting as an Isolated Colorectal Polyp‐Like Morphology
Source: JGH Open. 2025 Apr 30;9(5):e70170. doi: 10.1002/jgh3.70170 (PMC12043396; doi:10.1002/jgh3.70170)
Supplement: Supplementary file 1 — Table S1. Case series of colorectal follicular lymphoma presenting as a single, isolated polyp (English literature only). [file JGH3-9-e70170-s001.docx]

| **Supplementary table 1.** Case series of colorectal follicular lymphoma presenting as a single, isolated polyp (English literature only). | | | | | | | | | | | |
| --- | --- | --- | --- | --- | --- | --- | --- | --- | --- | --- | --- |
| **Case**  **number** | **Reference**  **number** | **Publish**  **year** | **Age  (years)** | **Sex** | **Clinical**  **symptoms** | **Lesion location** | **Lesion size**  **(mm)** | **Morphology** | **ER** | **Clinical**  **stage** | **Management** |
| 1 | [4] | 2010 | 74 | Male | FOBT positive | Descending colon | 35 | Pedunculated | Yes | I | ND |
| 2 | [5] | 2014 | 74 | Male | Thrombocytopenia | Transverse colon | ND | ND | Yes | I | Watch and wait |
| 3 | [6] | 2015 | 55 | Male | No symptom | Sigmoid colon | 10 | Sessile | Yes | I | ND |
| 4 | Current case | 2025 | 60 | Female | Hematochezia | Transverse colon | 8 | Sessile | Yes | I | Watch and wait |
| ER: Endoscopic resection, FOBT: fecal occult blood test, ND: Not described. | | | | | | | | | | | |
